# Association of metabolic comorbidity with myocardial infarction in individuals with a family history of cardiovascular disease: a prospective cohort study

**DOI:** 10.1186/s12889-022-14330-2

**Published:** 2022-10-31

**Authors:** Seokyung An, Sungji Moon, Sue K. Park

**Affiliations:** 1 Department of Biomedical Sciences, Seoul National University Graduate School, Seoul, Korea; 2 Department of Preventive Medicine, Seoul National University College of Medicine, Seoul, Korea; 3 Cancer Research Institute, Seoul National University, Seoul, Korea; 4 Interdisciplinary Program in Cancer Biology, Seoul National University College of Medicine, Seoul, Korea; 5 Integrated Major in Innovative Medical Science, Seoul National University College of Medicine, Seoul, Korea

**Keywords:** Family history of cardiovascular disease, Diabetes mellitus, Hypertension, Dyslipidemia, Comorbidity, Myocardial infarction

## Abstract

**Background:**

The association between metabolic comorbidity and myocardial infarction (MI) among individuals with a family history of cardiovascular disease (CVD) is yet to be elucidated. We aimed to examine the combined effects of metabolic comorbidities, including diabetes mellitus, hypertension, and dyslipidemia, with a family history of CVD in first-degree on the risk of incident MI.

**Methods:**

This cohort study consisted of 81,803 participants aged 40–89 years without a previous history of MI at baseline from the Korean Genome and Epidemiology Study. We performed Cox proportional hazard regression analysis to estimate the hazard ratios (HRs) and 95% confidence intervals (CIs) for MI and early-onset MI risk associated with metabolic comorbidity in individuals with a family history of CVD.

**Results:**

During a median follow-up of 5 years, 1,075 and 479 cases of total and early-onset MI were reported, respectively. According to the disease score, among individuals who had a positive family history of CVD, the HRs for MI were 1.92 (95% CI: 1.47–2.51) in individuals with one disease, 2.75 (95% CI: 2.09–3.61) in those with two diseases, and 3.74 (95% CI: 2.45–5.71) in those with three diseases at baseline compared to individuals without a family history of CVD and metabolic diseases. Similarly, an increase of the disease score among individuals with a positive family history of CVD was associated with an increase in early-onset MI risk.

**Conclusion:**

Metabolic comorbidity was significantly associated with an increased risk of MI among individuals with a family history of CVD.

**Supplementary Information:**

The online version contains supplementary material available at 10.1186/s12889-022-14330-2.

## Background

The prevalence of metabolic comorbidity, defined as having one or more metabolic diseases, is constantly increasing [1]. Metabolic diseases, including diabetes mellitus (DM), hypertension (HTN), and dyslipidemia (DLP), are the leading risk factors of myocardial infarction (MI), which is the major cause of death worldwide [2–4]. The prevalence of these diseases has been reported in more than half of the patients with MI [5]. Previous studies have also found that the combination of these diseases is significantly associated with an increased risk of cardiovascular disease (CVD) outcomes [6–8].

Another remarkable predictor of MI is a family history of CVD [9]. Moreover, middle-aged adults with a positive family history of CVD are strongly associated with a risk of MI [10, 11]. The relationship between metabolic comorbidity and MI may differ depending on the family history of CVD. Particularly, among individuals with family history of CVD, metabolic comorbidity can play an important role in the development of MI. However, there is limited evidence on the impact of comorbidity in this group.

Estimating the risk and predictors of MI is essential in developing preventive efforts for people who are at a high risk of MI. The purpose of this study is to examine the association between metabolic comorbidity and the risk of incident MI in relation to a family history of CVD.

## Methods

### Ethic statements

The Institutional Review Board of the Seoul National University approved this study protocol (number. 1912-063-1088), and informed consent was obtained from all participants. This study followed the Strengthening the Reporting of Observational Studies in Epidemiology (STROBE) statement for cohort studies [12].

## Data sources and study population

The study population was derived from the Korean Genome and Epidemiology Study (KoGES), including the Health Examinees study (HEXA), Cardiovascular Disease Association Study (CAVAS), the Ansan and Ansung study. The KoGES database was a community-based multicenter study consisting of participants aged ≥40 years who underwent a health examination. Baseline data were obtained between 2004 and 2013, 2005 and 2011, and 2001 and 2002 and follow-up data were obtained between 2012 and 2017, 2007 and 2014, and 2003 and 2014 from HEXA, CAVAS, and the Ansan and Ansung study, respectively. Information on participants’ socio-economic status, medical history, family history of disease, lifestyle factors, medication usage, and diet was collected though an interview-based questionnaire. Trained staffs obtained data from health examinations and laboratory blood tests. Details of the study design have been described previously [13].

**Fig. 1:**
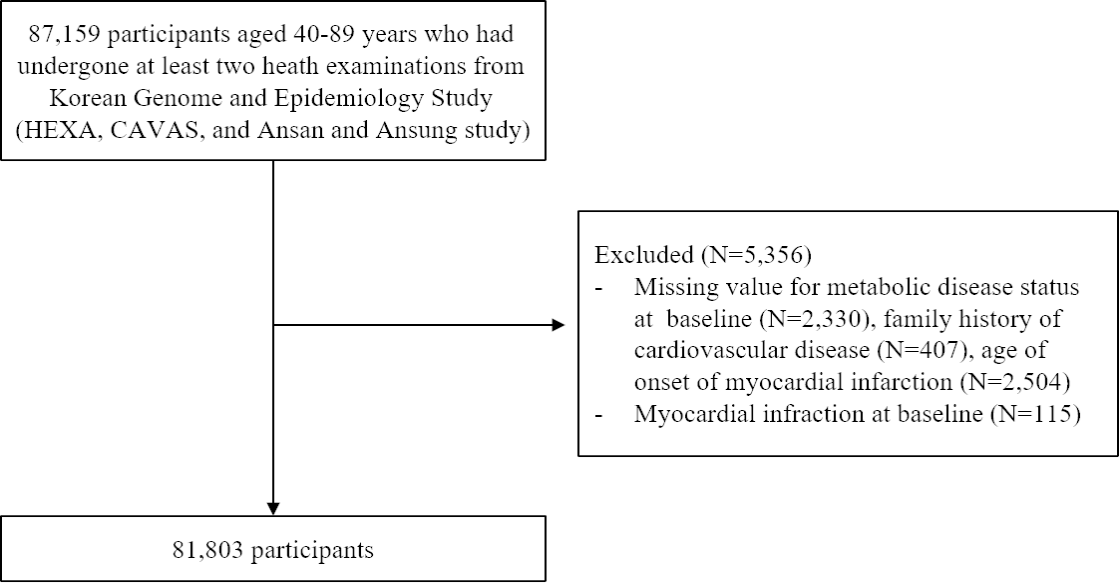
Flow chart of the study population selection from the Korean Genome and Epidemiology Study. Abbreviations: Health Examinees study (HEXA);  Cardiovascular Disease Association Study (CAVAS)

Among 87,159 individuals aged 40–89 years who had received at least two health examinations, 2,330, 407, and 2,504 participants without information on their metabolic disease status (HTN, DM, and DLP), family history of CVD, and age of onset of MI were excluded, respectively. An additional 115 participants who had MI at baseline or missing values on MI diagnosis were excluded. Finally, a total of 81,803 participants were included in the study (Fig. 1). [12]

## Definition of metabolic comorbidity and family history of CVD

DM was defined as a fasting glucose level ≥ 126 mg/dL, glycated hemoglobin A1c level ≥ 6.5%, anti-diabetic drugs uses [14], or a self-reported diagnosis of DM. HTN was defined as a blood pressure ≥ 130 mmHg, diastolic blood pressure ≥ 80 mmHg, antihypertensive drugs uses [15], or a self-reported diagnosis of HTN. DLP was defined as a total cholesterol level ≥ 240 mg/dL, triglyceride level ≥ 200 mg/dL, high-density lipoprotein cholesterol level < 40 mg/dL, lipid-lowering drug use [16], or a self-reported diagnosis of DLP. The disease score was calculated according to the presence of DM, HTN, and DLP at baseline. A self-reported diagnosis of family history of CVD in a first-degree was used to define a family history of CVD.

## Study outcomes

The primary outcome was a new diagnosis of non-fatal MI, which was defined as a positive response to self-reported medical history of MI at a follow-up examination. The study endpoint was the date of non-fatal MI diagnosis. Early-onset MI was defined as an incident occurring in men aged ≤ 55 years and in women aged ≤ 65 years [17]. The dates of the latest follow-up were February 2017 in HEXA and December 2014 in CAVAS and the Ansan and Ansung study.

### Statistical analysis

The baseline characteristics between individuals with negative and positive family histories of CVD were compared using the Student t-test and chi-square test for continuous and categorical variables, respectively. For continuous variables, quantile-quantile (Q-Q) plot, kurtosis, and skewness were used to assess normality [18]. We categorized participants into eight groups based on baseline disease status: (1) absence of DM, HTN, and DLP and presence of (2) DM, (3) HTN, (4) DLP, (5) DM and HTN, (6) DM and DLP, (7) HTN and DLP, and (8) DM, HTN, and DLP. The number of conditions was used to calculate the disease score. We performed multivariable Cox proportional hazards regression analysis to estimate hazard ratios (HRs) with 95% confidence intervals (CIs) for MI according to a family history of CVD and the baseline disease status. To assess the fitness of the Cox proportional hazard model, proportional hazard assumption was evaluated with scaled Schoenfeld residuals.

For analysis, we assessed a combined association between metabolic comorbidity and a family history of CVD and MI. Adjusted HRs and 95% CI for MI were calculated from adjusting the potential confounding factors including age, sex (male and female), body mass index (< 25 and ≥25 kg/m^2^), waist-to-hip ratio (< 0.90 or ≥ 0.90 for male and < 0.85 or ≥ 0.85 for female), income level (<$2,000, $2,000–4,000, and ≥$4,000 per month), smoking status (never, past, and current smoker), alcohol drinking status (never, past, and current drinker), and regular exercise (yes and no). In this analysis, we considered individuals with a negative family history of CVD and none of the metabolic diseases as the reference group. All statistical analyses were conducted using SAS 9.4 software (SAS Institute, Cary, NC, USA) and R (version 4.0.5.), and a *P-*value < 0.05 was considered statistically significant.

## Results

Among 81,803 individuals, 15,754 (19.3%) had a family history of CVD in a first-degree, whereas 66,049 (80.7%) reported a negative family history of CVD. At baseline, individuals with a positive family history of CVD were more likely to be current alcohol drinkers and to have HTN and DLP compared to the those with a negative family history of CVD (Table 1).

**Table 1:** Baseline characteristics of participants by family history of cardiovascular disease

	Negative family history of CVD (N = 66,049)	Positive family history of CVD (N = 15,754)	***p***-value
Age, years	54.2 ± 8.58	53.9 ± 8.15	< 0.001
Male, N (%)	23,950 (36.3)	5,294 (33.6)	< 0.001
Current smoker, N (%)	94,98 (14.4)	2,023 (12.8)	< 0.001
Current alcohol drinker, N (%)	28,950 (43.8)	7,023 (44.6)	0.034
Regular exercise, N (%)	33,857 (51.3)	8,448 (53.6)	< 0.001
BMI ≥ 25 kg/m^2^	22,577 (24.2)	5,493 (34.9)	0.090
WHR ≥ 0.90 for men, 0.85 for women	31,293 (47.4)	7,323 (46.5)	0.053
Monthly income ≥ $4,000 K, N (%)	11,674 (17.7)	3,434 (21.8)	< 0.001
Hypertension, N (%)	35,800 (54.2)	9,121 (57.9)	< 0.001
Diabetes mellitus, N (%)	6,587 (9.9)	1,501 (9.5)	0.093
Dyslipidemia, N (%)	25,063 (37.9)	6,402 (40.6)	< 0.001

Abbreviation, Cardiovascular disease (CVD); Number (N); Body mass index (BMI); Waist to hip ratio (WHR);

During a median follow-up of 5 years (range, 1–18 years), there were 1,075 (1.3%) and 479 (0.6%) cases of MI and early-onset MI, respectively. Compared to individuals with a negative family history of CVD, those with a positive family history showed a greater risk for MI (HR 1.28, 95% CI: 1.11–1.48). The risks of MI were 1.58 (95% CI: 1.31–1.91) in participants with one disease, 2.11 (95% CI: 1.73–2.58) in those with two diseases, and 2.52 (95% CI: 1.92–3.32) in those with three diseases. Similarly, an increase of the disease score was associated with an increase in early-onset MI risk (Supplementary Table 1).

The combined association of a family history of CVD and metabolic diseases with the risk of MI is shown in Table 2. After adjustment for age, sex, body mass index, waist to hip ratio, income level, smoking status, alcohol drinking, and regular exercise, individuals with a positive family history and metabolic disease had a higher risk of MI and early-onset MI than the reference group. Among individuals who had a positive family history of CVD, the adjusted HRs for MI were 1.32 (95% CI: 0.89–1.95) for participants with none of the diseases, 1.38 (95% CI: 0.34–5.58) in those with DM, 2.03 (95% CI: 1.51–2.74) in those with HTN, 1.68 (95% CI: 1.07–2.66) in those with DLP, 1.98 (95% CI: 0.96–4.06) in those with DM and HTN, 3.24 (95% CI: 1.19–8.79) in those with DM and DLP, 2.90 (95% CI: 2.18–3.86) in those with HTN and DLP, and 3.74 (95% CI: 2.45–5.71) in those with DM, HTN, and DLP compared to those with a negative family history of CVD and none of the metabolic diseases (Table 2). Individuals with a positive family history of CVD, DM and DLP (HR 7.36, 95% CI: 2.30-23.52), HTN and DLP (HR 3.69, 95% CI: 2.43–5.62), and DM, HTN, and DLP at baseline (HR 6.73, 95% CI: 3.59–12.63) had a significantly increased risk for early-onset MI (Table 2). The associations were similar between individuals with and without a family history of CVD (MI, *P* for interaction = 0.801 and early-onset MI, 0.701).

**Table 2:** Combined association of family history of cardiovascular disease and combination of metabolic disease with myocardial infarction risk

Family history of CVD	No. of participants	Myocardial infarction				Early-onset myocardial infraction		
		**No. of MI**	**Hazard Ratio** ^**1**^ **(95% CI)**	**Hazard Ratio** ^**2**^ **(95% CI)**	**No. of early-onset MI** ^**1**^		**Hazard Ratio** ^**1**^ **(95% CI)**	**Hazard Ratio** ^**2**^ **(95% CI)**
**Negative**								
None	20,002	117	1.00	1.00	68		1.00	1.00
DM	945	17	3.10 (1.86–5.16)	2.09 (1.25–3.48)	4		1.27 (0.46–3.47)	1.55 (0.56–4.25)
HTN	18,095	237	2.27 (1.82–2.83)	1.59 (1.27–1.99)	109		1.80 (1.33–2.43)	2.09 (1.54–2.86)
DLP	8,357	94	1.99 (1.51–2.61)	1.60 (1.22–2.10)	46		1.67 (1.15–2.43)	1.86 (1.28–2.72)
DM and HTN	1,944	46	4.17 (2.96–5.86)	2.27 (1.60–3.22)	16		2.52 (1.46–4.34)	3.29 (1.88–5.76)
DM and DLP	945	24	4.54 (2.93–7.04)	2.86 (1.83–4.45)	3		0.99 (0.31–3.13)	1.25 (0.39–3.99)
HTN and DLP	13,008	232	3.19 (2.56–3.99)	2.02 (1.61–2.55)	79		1.88 (1.36–2.60)	2.32 (1.65–3.27)
DM, HTN, and DLP	2,753	65	4.37 (3.23–5.92)	2.58 (1.90–3.50)	27		3.13 (2.00-4.89)	4.11 (2.57–6.57)
**Positive**								
None	4,354	31	1.23 (0.83–1.83)	1.32 (0.89–1.95)	20		1.37 (0.83–2.25)	1.32 (0.80–2.17)
DM	176	2	1.99 (0.49–8.04)	1.38 (0.34–5.58)	2		3.41 (0.84–13.9)	3.95 (0.97–16.13)
HTN	4,401	67	2.70 (2.00-3.65)	2.03 (1.51–2.74)	37		2.57 (1.72–3.83)	2.88 (1.92–4.32)
DLP	1,943	22	2.01 (1.27–3.16)	1.68 (1.07–2.66)	15		2.35 (1.34–4.11)	2.49 (1.42–4.37)
DM and HTN	421	8	3.48 (1.70–7.13)	1.98 (0.96–4.06)	3		2.26 (0.71–7.17)	2.96 (0.93–9.48)
DM and DLP	160	4	4.64 (1.71–12.6)	3.24 (1.19–8.79)	3		5.98 (1.88–18.99)	7.36 (2.30-23.52)
HTN and DLP	3,555	84	4.35 (3.29–5.76)	2.90 (2.18–3.86)	35		3.13 (2.08–4.70)	3.69 (2.43–5.62)
DM, HTN, and DLP	744	25	6.40 (4.15–9.85)	3.74 (2.45–5.71)	12		5.30 (2.87–9.79)	6.73 (3.59–12.63)

Abbreviation, Cardiovascular disease (CVD); Myocardial infarction (MI); Confidence interval (CI); Hypertension (HTN); Diabetes mellitus (DM); Dyslipidemia (DLP)

1. Unadjusted hazard ratios.

2. Adjusted by sex, age at baseline, body mass index, waist and hip ratio, income level, current smoking status, current alcohol drinking, and regular exercise.

**Fig. 2:**
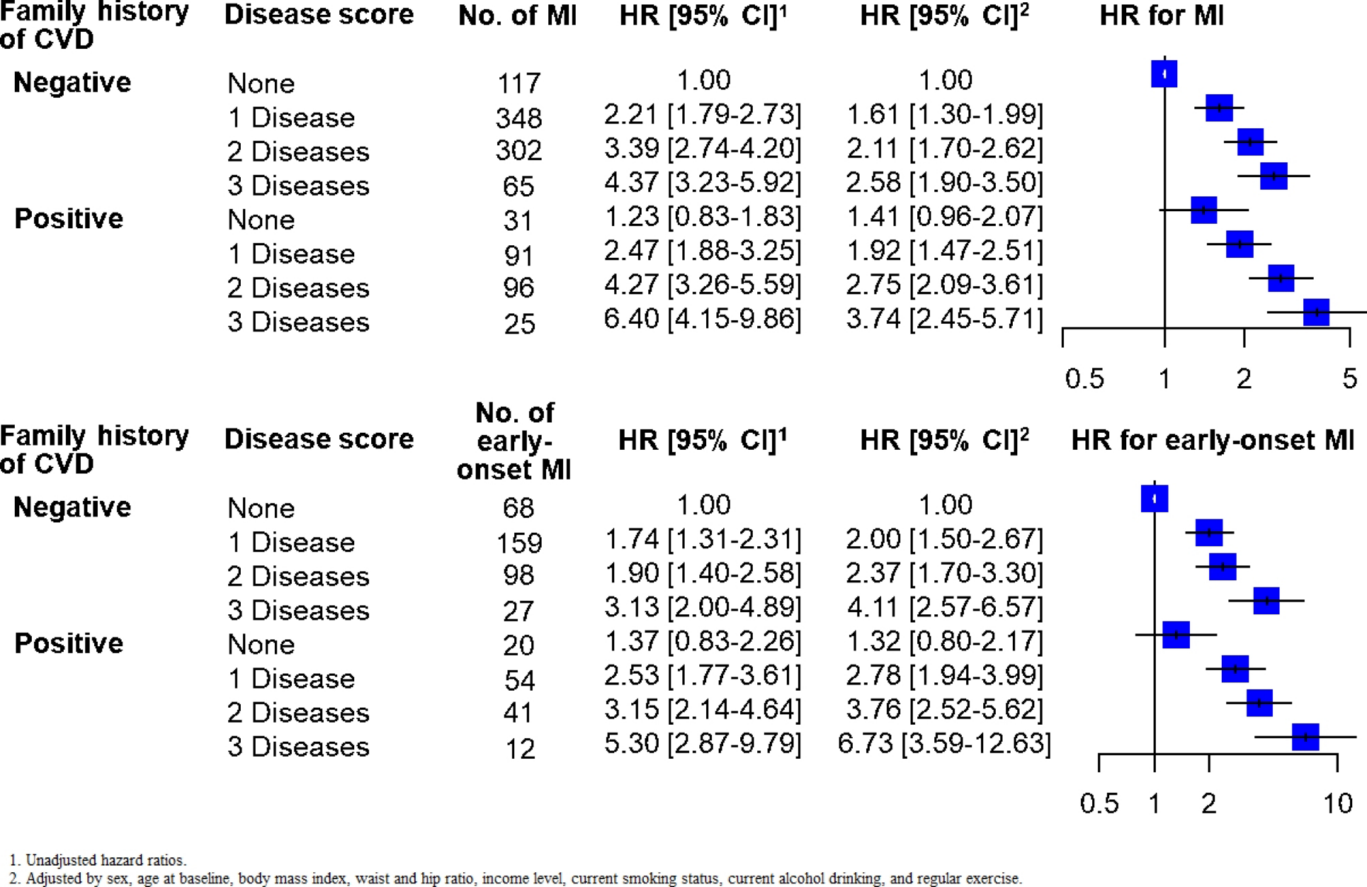
Combined association of family history of cardiovascular disease and disease score with overall and early-onset myocardial infarction risk. Abbreviation: Cardiovascular disease (CVD); Myocardial infarction (MI); Hazard ratio (HR); Confidence interval (CI)

According to the disease score, among people with a positive family history of CVD, the HRs for MI were 1.92 (95% CI: 1.47–2.51) in individuals with one disease, 2.75 (95% CI: 2.09–3.61) in those with two diseases, and 3.74 (95% CI: 2.45–5.71) in those with three diseases (Fig. 2). For early-onset MI, the HRs were 2.78 (95% CI: 1.94–3.99) in individuals with a positive history of CVD and one disease, 3.76 (95% CI: 2.52–5.62) in those with two diseases, and 6.73 (95% CI: 3.59–12.63) in those with three diseases. The similar association was shown between individuals with and without a family history of CVD (MI, *P* for interaction = 0.754 and early-onset MI, 0.904). The risk for MI and early-onset MI significantly increased with an increasing number of metabolic diseases (*P* for trend < 0.001) (Fig. 2).

## Discussion

In our study, we found that among individuals with a family history of CVD, the coexistence of DM, HTN, and DLP was associated with a 3.74-fold increased risk of MI and a 6.73-fold increased risk of early-onset MI compared to the absence of a family history of CVD and the metabolic diseases. Our study results demonstrated that metabolic comorbidity was associated with a high risk of MI among people with a family history of CVD.

Metabolic disease was associated with an increased risk of MI, as demonstrated in our study. With the aging population, the prevalence of metabolic comorbidity is constantly increasing, and a continued increase in CVD is inevitable [4, 19, 20]. Our results are in close agreement with those of previous studies that examined the impact of HTN and DLP on the future CVD risk in diabetic patients [6, 7]. [21–23]However, few studies have investigated the additive associations of a combination of multiple metabolic comorbidities with the risk of incident MI, particularly early-onset MI.

DM, HTN, and DLP are important independent risk factors for CVD and are used as major clinical variables for the prediction of CVD [2–4, 24]. The main pathophysiology of acute MI consists of plaque rupture in the coronary artery [25]. Metabolic diseases commonly generate reactive oxygen species [26–29], which promote the formation and progression of atherosclerotic plaques [30, 31], which can eventually become unstable and rupture [2, 32]. As each metabolic comorbidity accumulates, the oxidative stress in the coronary artery is aggravated [29], leading to higher risk of atherosclerosis and its rupture resulting in MI.

A family history of CVD is another major risk factor for MI [33]. Previous studies have reported that family history represents a genetic predisposition that contributes to an increased risk of MI [34], which is in line with our results. Early-onset CVD is strongly related to genetic susceptibility compared to late-onset CVD [11, 35]. Moreover, positive parental history of CVD is associated with a greater risk of metabolic disease prevalence than a negative parental history of CVD [36]. However, no prior study has found a relationship between metabolic comorbidity and MI events in patients with a family history of CVD. Our study identified individuals with metabolic comorbidity who were at a high risk of overall and early-onset MI based on their genetic background. Our results suggested, for the first time, that metabolic comorbidity contributed to familial aggregation of MI. Future genetic and environmental interactions studies are important to support our findings and provide individualized prevention strategies. However, our study’s findings should be interpreted in the context of some limitations. First, as our study used self-reported history of disease, family history of CVD, and outcomes, there was misclassification bias, which could have underestimated or overestimated the values. Previous validation studies, however, reported that the accuracy of both self-reported family history of CVD was > 80% [37, 38]. Since any fatal MI would be lost to follow up in this study, further studies confirming the diagnosis of metabolic diseases and MI using validated International Classification of Diseases codes [39] will be required to support this association. Second, there may be potential selection bias due to the study design of non-routine health examinations. Third, our study included only Korean participants. As genetic predisposition is diverse according to different ethnicities [33], further studies should be conducted to establish the relationship between family history and the CVD risk across ethnicities on a global level. Despite these limitations, our study was conducted based on a large sample size and prospective design with a long follow-up period. To our knowledge, our study is the first to estimate the effects of metabolic comorbidity on MI among individuals with a family history of CVD.

## Conclusion

In conclusion, our study found that metabolic comorbidity was significantly associated with an increased risk of MI among individuals who had a family history of CVD. Our study highlights the necessity of accounting for metabolic comorbidity among high-risk individuals to reduce the risk of MI.

## Electronic supplementary material

Below is the link to the electronic supplementary material.

Supplementary Material 1

## Data Availability

This article is based on data from the Korean Genome and Epidemiology Study (KoGES). The governmental index of KoGES data is publicly contactable, via https://nih.go.kr/contents.es?mid=a50401010400. The datasets generated and/or analyzed during the current study are available from the corresponding author on reasonable request.

## Abbreviations

DM: Diabetes mellitus
HTN: Hypertension
DLP: Dyslipidemia
MI: Myocardial infarction
CVD: Cardiovascular disease
KoGES: Korean Genome and Epidemiology Study
HEXA: Health Examinees Study
CAVAS: Cardiovascular Disease Association Study
HR: Hazard ratio
CI: Confidence interval
