# Supplementary material for: Association of metabolic comorbidity with myocardial infarction in individuals with a family history of cardiovascular disease: a prospective cohort study
Source: BMC Public Health. 2022 Oct 31;22:1992. doi: 10.1186/s12889-022-14330-2 (PMC9624008; doi:10.1186/s12889-022-14330-2)
Supplement: Supplementary file 1 — Supplementary Material 1 [file 12889_2022_14330_MOESM1_ESM.docx]

Supplementary Table 1. Association between metabolic disease status at the baseline and the risk of myocardial infraction in total population

| **Characteristics** | **No. of**  **participants** | **Myocardial infarction** | | | **Early-onset myocardial infraction** | | |
| --- | --- | --- | --- | --- | --- | --- | --- |
|  |  | **No. of MI** | **Hazard Ratio^1^**  **(95% CI)** | **Hazard Ratio^2^**  **(95% CI)** | **No. of early-onset MI^1^** | **Hazard Ratio^1^**  **(95% CI)** | **Hazard Ratio^2^**  **(95% CI)** |
| **Family history of CVD** |  |  |  |  |  |  |  |
| No | 66,049 | 832 | 1.00 | 1.00 | 352 | 1.00 | 1.00 |
| Yes | 15,754 | 243 | 1.25 (1.08-1.44) | 1.28 (1.11-1.48) | 127 | 1.54 (1.26-1.89) | 1.46 (1.19-1.79) |
| **DM** |  |  |  |  |  |  |  |
| No | 73,715 | 884 | 1.00 | 1.00 | 409 | 1.00 | 1.00 |
| Yes | 8,088 | 191 | 2.04 (1.74-2.38) | 1.37 (1.17-1.61) | 70 | 1.62 (1.26-2.09) | 1.58 (1.22-2.06) |
| **HTN** |  |  |  |  |  |  |  |
| No | 36,882 | 311 | 1.00 | 1.00 | 161 | 1.00 | 1.00 |
| Yes | 44,921 | 764 | 2.06 (1.81-2.36) | 1.39 (1.21-1.59) | 318 | 1.66 (1.38-2.01) | 1.75 (1.43-2.15) |
| **DLP** |  |  |  |  |  |  |  |
| No | 50,338 | 525 | 1.00 | 1.00 | 259 | 1.00 | 1.00 |
| Yes | 31,465 | 550 | 1.74 (1.55-1.96) | 1.35 (1.19-1.53) | 220 | 1.41 (1.18-1.69) | 1.36 (1.13-1.65) |
| **Combination of disease** |  |  |  |  |  |  |  |
| None | 24,356 | 148 | 1.00 | 1.00 | 88 | 1.00 | 1.00 |
| DM | 1,122 | 19 | 2.81 (1.75-4.54) | 1.89 (1.17-3.05) | 146 | 1.50 (0.66-3.44) | 1.85 (0.81-4.24) |
| HTN | 22,496 | 304 | 2.26 (1.85-2.75) | 1.58 (1.29-1.94) | 6 | 1.83 (1.40-2.38) | 2.11 (1.61-2.77) |
| DLP | 10,300 | 116 | 1.91 (1.50-2.44) | 1.53 (1.20-1.95) | 61 | 1.69 (1.22-2.34) | 1.87 (1.34-2.60) |
| DM and HTN | 2,365 | 54 | 3.30 (2.72-4.01) | 2.11 (1.53-2.91) | 19 | 2.32 (1.41-3.81) | 3.07 (1.84-5.13) |
| DM and DLP | 1,105 | 28 | 4.37 (2.92-6.55) | 2.79 (1.85-4.20) | 114 | 1.59 (0.70-3.64) | 2.05 (0.89-4.73) |
| HTN and DLP | 16,563 | 316 | 3.89 (2.85-5.31) | 2.07 (1.69-2.54) | 6 | 2.01 (1.52-2.65) | 2.44 (1.81-3.29) |
| DM, HTN, and DLP | 3,497 | 90 | 4.61 (3.54-5.99) | 2.52 (1.92-3.32) | 39 | 3.36 (2.31-4.91) | 4.37 (2.92-6.54) |
| **Disease score** |  |  |  |  |  |  |  |
| None | 24,356 | 148 | 1.00 | 1.00 | 88 | 1.00 | 1.00 |
| 1 disease | 33,917 | 439 | 2.17 (1.80-2.62) | 1.58 (1.31-1.91) | 213 | 1.78 (1.39-2.28) | 2.02 (1.57-2.61) |
| 2 diseases | 20,033 | 398 | 3.43 (2.84-4.14) | 2.11 (1.73-2.58) | 139 | 2.02 (1.55-2.64) | 2.48 (1.86-3.31) |
| 3 diseases | 3,497 | 90 | 4.61 (3.54-5.99) | 2.52 (1.92-3.32) | 39 | 3.36 (2.31-4.91) | 4.36 (2.91-6.52) |

Abbreviation, Hypertension (HTN); Diabetes mellitus (DM); Dyslipidemia (DLP)

1. Unadjusted hazard ratio
2. Adjusted by sex, age at baseline, body mass index, waist and hip ratio, current smoking status, current alcohol consumption, regular exercise, and family history of cardiovascular disease
